# Abnormal resting-state functional connectivity in the orbitofrontal cortex of heroin users and its relationship with anxiety: a pilot fNIRS study

**DOI:** 10.1038/srep46522

**Published:** 2017-04-19

**Authors:** Hada Fong-ha Ieong, Zhen Yuan

**Affiliations:** 1 University of Macau, Faculty of Health Sciences, Bioimaging Core, Macau SAR, 99999, China

## Abstract

Drug addiction is widely linked to the orbitofrontal cortex (OFC), which is essential for regulating reward-related behaviors, emotional responses, and anxiety. Over the past two decades, neuroimaging has provided significant contributions revealing functional and structural alternations in the brains of drug addicts. However, the underlying neural mechanism in the OFC and its correlates with drug addiction and anxiety still require further elucidation. We first presented a pilot investigation to examine local networks in OFC regions through resting-state functional connectivity (rsFC) using functional near-infrared spectroscopy (fNIRS) from eight abstinent addicts in a heroin-dependent group (HD) and seven subjects in a control group (CG). We discovered that the HDs manifested enhanced interhemispheric correlation and rsFC. Moreover, small-worldness was explored in the brain networks. In addition to the altered rsFC in the OFC networks, our examinations demonstrated associations in the functional connectivity between the left inferior frontal gyrus and other OFC regions related to anxiety in the HDs. The study provides important preliminary evidence of the complex OFC networks in heroin addiction and suggests neural correlates of anxiety. It opens a window in application of fNIRS to predict psychiatric trajectories and may create new insights into neural adaptations resulting from chronic opiate intake.

Drug addiction is a neuropsychiatric disorder characterized by compulsive drug-seeking and drug-taking behaviors despite adverse consequences^1^. As one of the most frequently abused drugs, heroin is widely recognized to be associated with dire societal consequences, undermined industrial and social advancement, increased crime, violence, insecurity and the spread of transmitted diseases like HIV or hepatitis C virus^2^,^3^,^4^. As a result, identifying the neurobiological mechanisms underlying heroin addiction will have substantial implications for the medical, psychological and legal management of drug addicts, as well as for the fundamental understanding of addictive behaviors.

In a normal brain, the orbitofrontal cortex (OFC), as part of the prefrontal cortex (PFC), plays an essential role in executive functions and inhibitory decision making, especially in the formulation and use of outcome expectancies related to reward^5^. The OFC mediates environmental stimuli, affective information, and emotional and social responses, such as demonstrating empathy, acessesing one’s own and other individuals’ thoughts and predicting future consequnces and actions^6^. In contrast, well-documented neuroimaging studies of substance use disorders have provided accumulating evidence that disregulations in the decision-making functions of the OFC of an addict’s brain are closely associated with addictive traits, such as craving, salience, repeat drug use, and relapse^1^,^7^,^8^,^9^.

While task-based neuroimaging studies have provided convincing evidence that acute and/or chronic exposure to drugs of abuse can cause both short- and long-term consequences within delineated brain areas as well as essential insights into the neuroplastic adaptations resulting from drug exposure, these findings have failed to consistently described substantial brain activities in the OFC and their relationship to a particular drug. For example, previous studies attempting to detect different OFC activations in patients with methamphetamine^10^,^11^ and cocaine^12^ use disorders, both involving stimulants, these findings were not consistent. Paulus *et al*.^10^,^11^ demonstrated decreased task-related activity in the right OFC of methamphetamine users, whereas Bolla *et al*.^12^ reported increased brain activity in cocaine users. A more comprehensive strategy is needed to explain the underlying neural mechanisms in addiction to various drugs. The type of drug of abuse, the stage of the abuser, and other essential elements, such as comorbid anxiety or depression, and co-dependence on other substances, such as nicotine, should be considered and accounted in studies in order to better understand the essentiality of reward, anticipation, and cognition in the OFC networks. This may ultimately provide tools to predict relapse in individuals trying to recover from addiction.

One previous study found that anxiety can be a predictor of relapse^13^. Recently, an additional study^14^ demonstrated that anxiety produced by environmental stressors played an essential role in the connectivity among the OFC, inferior frontal gyrus, ventromedial PFC, and amygdala; this anxiety was linked to a range of impaired goal-directed psychiatric disorders, such as addiction. Drug addiction involves deficits in controlling drug-seeking and drug-taking behaviors via inhibitory decision making^8^ – a function that presumably depends on connectivity within the OFC cortical network. Thus, in the present study we hypothesized that alterations in the OFC correlated with anxiety in heroin addiction.

Recently, there has been growing evidence that resting-state functional connectivity (rsFC) can provide significant insight and may be a biomarker for the early detection of impaired neural circuitry underlying diverse neuropsychiatric disorders^15^,^16^,^17^. The theory is that neurons synchronized at rest are synaptically bound to defined, straightforward, or multiple interconnected regions^18^,^19^,^20^ and therefore create credible and logical functional networks^17^,^21^. Furthermore, the strength of these functional networks at rest can predict accurate behavioral responses and the sequential arousal of the same brain regions when tasks are being performed^17^,^22^,^23^.

More importantly, functional near-infrared spectroscopy (fNIRS), a rapidly growing non-invasive neuroimaging technique, offers good temporal resolution and provides quantitative hemodynamic information regarding both oxyhemoglobin (HbO) and deoxyhemoglobin (Hb)^24^,^25^,^26^,^27^. fNIRS has been implemented in the form of a wearable device capable of intuitively monitoring brain activity in real-life conditions^28^, it has shown promise over the past few decades to support rsFC studies in both healthy individuals^27^ and psychiatric patients^15^,^16^ with depression^29^, attention deficit/hyperactive disorder^30^, and autism^31^, suggesting that alterations in rsFC strength has potential as a biomarker to examine different psychiatric disorder trajectories. Recently, the use of fNIRS to assess functional connectivity has been extended to psychiatric patients with arachnophobia^32^ and children with psychopathology risk^33^.

To the best of our knowledge, no investigations have been conducted using fNIRS measurements to identify the OFC activation network patterns and the rsFC of individuals with heroin addiction. Although this pilot study is not sufficient to provide full verification of the above stated hypotheses, the objectives of our experimental investigation are multi-fold: (1) to explore the neural networks generated within and between the particular OFC brain regions when an individual with heroin addiction is *at rest;* (2) to examine whether rsFC in the OFC is increased or decreased by focusing on abstinent heroin addicts; and (3) to investigate correlations with anxiety in local OFC networks. This pilot study used resting-state fNIRS in a small sample of abstinent heroin-dependent (HD) individuals in order to identify novel neural correlates of opioid addiction and anxiety in the OFC system.

## Results

### Interhemispheric correlations

**Figure 1:**
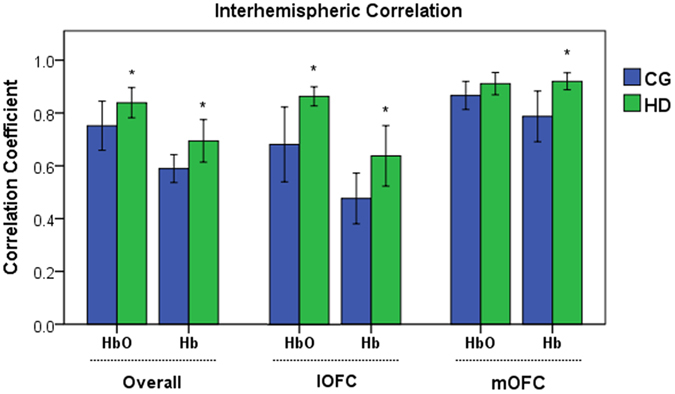
Interhemispheric correlations in the lateral OFC (lOFC) and medial OFC (mOFC) of abstinent heroin-dependent subjects (HDs, green, n = 8) and control subjects (CGs, blue, n = 7). Error bars represent the standard error of the mean over all the subjects. Compared with the CGs, the HDs demonstrated significantly increased interhemispheric HbO correlation values overall (*p* = *0.016*) with the rest of the channels and the lOFC (*p* = *0.033*) as well as greater Hb correlation values overall (*p* = *0.012*) with the rest of the channels, lOFC (*p* = *0.049*), and mOFC (*p* = *0.012*).

The results of the interhemispheric correlation analysis are shown in Fig. 1. The HbO and Hb signals were examined to explore the long-distance brain connectivity through the intravascular events^34^ between the hemispheres. Compared with the control group subjects (CGs), the study group (HDs) manifested enhanced overall functional connectivity between the left and right hemispheres, including all the channels that covered the lOFC and mOFC regions (see Supplementary Table S2). Specifically, the results of Student’s *t*-test showed that, compared with the CGs, the HDs exhibited significantly higher overall left-right correlation values for HbO (*r*_HD_ = 0.857 ± 0.024, *r*_CG_ = 0.751 ± 0.046, *p* = 0.016) and significantly higher HbO values in the lOFC (*r*_HD_ = 0.871 ± 0.017, *r*_CG_ = 0.678 ± 0.070, *p* = 0.033). More importantly, the HDs also showed significantly greater overall interhemispheric correlation values for Hb than the CGs (*r*_HD_ = 0.727 ± 0.039, *r*_CG_ = 0.590 ± 0.027, *p* = 0.012) and in the mOFC (*r*_HD_ = 0.919 ± 0.016, *r*_CG_ = 0.787 ± 0.047, *p* = 0.012) and lOFC regions (*r*_HD_ = 0.625 ± 0.050, *r*_CG_ = 0.476 ± 0.047, *p* = 0.049). In contrast, the HbO values in the mOFC regions (*p* = 0.206) did not show a significant difference between the groups.

### Seed-based localizer results

For simplicity, the HbO signals from all the channels across all the subjects were analyzed and reported in the remainder of the study. Seed channels were selected primarily based on brain activation in the gambling localizer task. Among all the channels covering the OFC regions, channel 11 in the left hemisphere and channel 4 in the right hemisphere were identified to have the most notable gambling task-induced HbO changes. Therefore, with symmetric localization, the OFC seed channels for rsFC analysis were selected and localized at channels 11 and 12 for the left hemisphere and channels 4 and 1 for the right hemisphere. To illustrate resting-state low-frequency fluctuations, resting-state time courses from the seed channels (i.e., channels 11 and 4) of one subject from each group are displayed in Supplementary Figure S3.

### Functional connectivity measurement: seed-based correlation results

**Figure 2:**
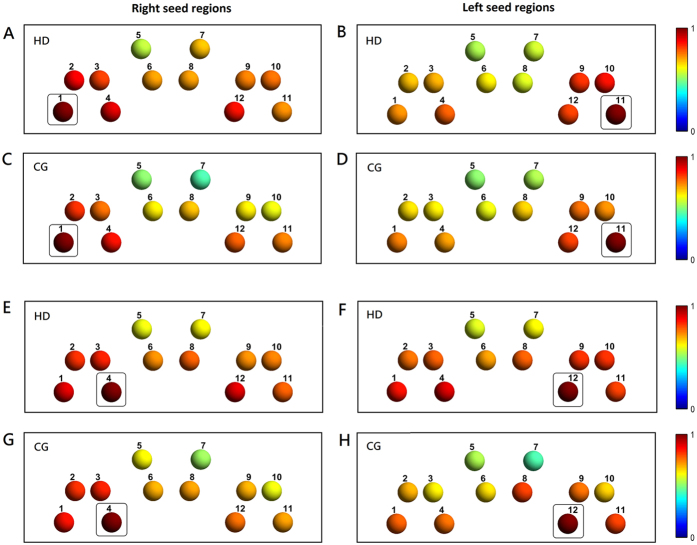
Seed-based rsFC Z-R maps of the OFC systems. (**A**,**B**) and (**E**,**F**) represent the HDs. (**C**,**D**) and (**G**,**H)** represent the CGs. The left column indicates the right seed channel OFC regions, and the right column indicates the left seed channel OFC regions. Channels that had enhanced rsFC with OFC seed regions are displayed in warm colors. The selected seed channels are boxed. The seed channels were selected based on the localizer task activation seed channels (i.e., channel 11 with its symmetrical channel 12 for the left side and channel 4 with its symmetrical channel 1 for the right). Each channel is numbered. Color bars represent the HbO-based Z to R correlation coefficient values.

Group-level results of seed-based rsFC maps are shown in Fig. 2. By estimating the strength of pairwise relationships between the selected seed channels and the channels covering other OFC regions for both the HDs and CGs, seed-based rsFCs were observed *between* bilateral and *within* ipsilateral OFC regions. Bilaterally to the right seed regions, considerable correlations in the left regions were shown in channels 10–12 (covering the contralateral OFC regions) in the HDs.

Ipsilaterally within the left seed regions, a considerable correlation was observed in channel 10 in the HDs but not in the CGs. Interestingly, for seed channel 12, both groups displayed a considerable correlation with ipsilateral channel 8, covering the left mOFC, but not for seed channel 11.

### Functional connectivity measurement: whole-brain correlation results

**Figure 3:**
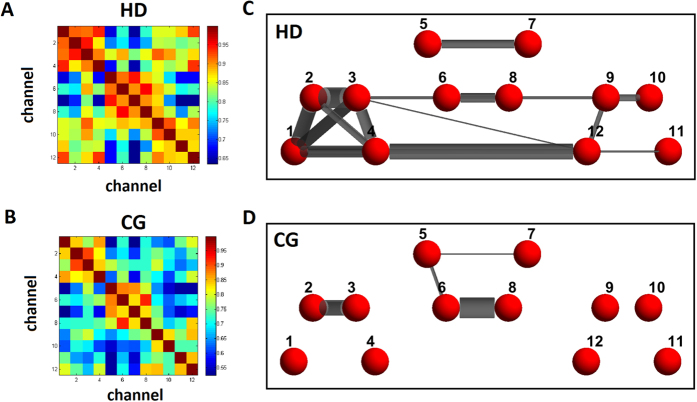
Whole-brain correlation analysis for comparison of the brain networks between the HDs and CGs. (**A**,**B**) Connectivity matrices of the HDs and CGs, respectively. Color bars represent the correlation coefficient values. (**C**,**D**) Brain network patterns of the HDs and CGs, respectively. Only the topmost 5% with correlation values greater than 0.87 are shown in the figures. The nodes (red; channels) are numbered by channel, and the weighted edges (gray) are displayed.

Using resting-state fNIRS data, a mean group-level correlation matrix was obtained in the CGs, and its network topological attributes were compared with the HDs. Before generating network topological properties, we used the foremost 5% of the connections^35^ (correlation coefficient values >0.87; mean correlation = 0.75) calculated in the CGs (method details in Niu *et al*.^36^) because this allowed us to examine the absolute network organization of the controls for comparison with the study group, the HDs. Group-level HbO-based connectivity patterns for both the HDs and CGs by whole-brain analysis are shown in Fig. 3, where the nodes represent the channels and the edges represent the connectivity between the nodes. The HD and CG connectivity matrices are shown in Fig. 3A and B, respectively. The HD brain networks (Fig. 3C) demonstrated more edges in the OFC system than the CG brain networks (Fig. 3D) overall, but not within mOFC regions. Though, the HD network showed an especially strong connection between channels 6 and 8, which covered the right and left mOFCs, respectively.

**Figure 4:**
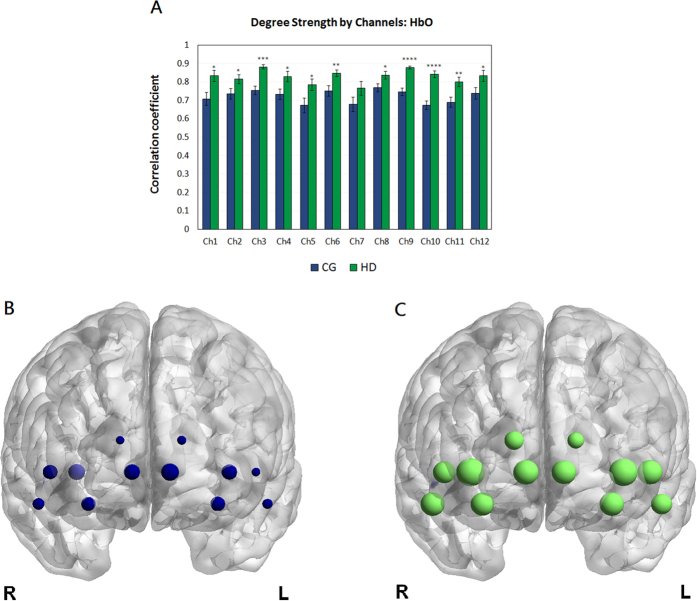
Connectivity strength by channel. (**A**) Connectivity strength with the group average of the correlation coefficients in OFC of the HDs (green) and CGs (blue). Error bars indicate the standard error of the mean across the HDs and CGs. Compared with the CGs, the HDs showed significantly greater connectivity strength in almost all the channels, except channel 7, in terms of HbO. (**B**,**C**) The size of each node indicates its corresponding nodal strength in the networks of two groups (CG, blue; HD, green). The strength values are shown in Supplementary Table S4. *p < 0.05; **p < 0.01; ***p < 0.001; ****p < 0.0001.

Next, we quantified the connectivity strength, *σ*, which is the average connectivity of a node in each network in the OFC system. We calculated the column mean of the HbO-based connectivity matrices, and group results are shown in Fig. 4. Group-level analysis demonstrates that the rsFC strengths in the HDs are remarkably (*p* < 0.001) higher in channels 9, 10, and 3. Channels 1, 2, 4, 5, 6, 8, 11, and 12 in the HDs also demonstrate greater connectivity strengths than in the CGs (see Supplementary Table S4).

### Mapping the small-world properties of brain networks in heroin addiction

**Figure 5:**
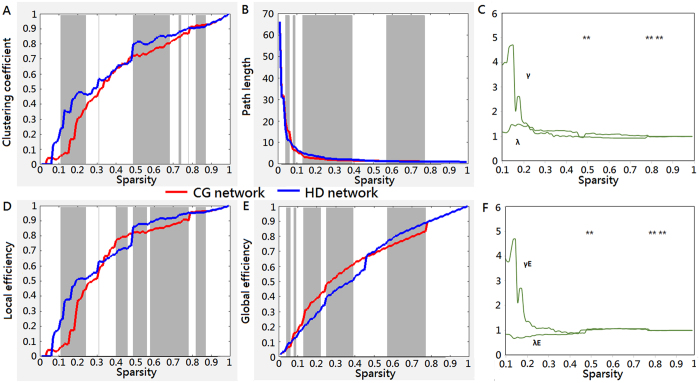
Comparison of the HD brain network to the CG brain network: Small-worldness and network efficiency. Network parameters were based on the functional networks generated from the HbO measurements over a wide range of sparsity. (**A**) Clustering coefficient, (**B**) characteristic path length, (**D**) local efficiency, and (**E**), global efficiency, were the network properties under study. For the entire range of sparsity thresholds from 0 to 1, the brain network in the HDs (blue) had greater clustering coefficients and local efficiency values than the brain network in the CGs (red). Normalized clustering coefficients (γ, green) and normalized path lengths (λ, green) with respect to the CG network are shown in (**C)**. Normalized local efficiency (γE, green) and normalized global efficiency (λE, green) with respect to the CG network are shown in (**F**). The normalized path length and global efficiency values in both groups shared similar patterns, resulting in γ and γE > 1 and λ and λE = ~1. These findings may indicate that the rsFC networks in the HDs carried small-world characteristics with respect to the CG networks. The gray zones represent a significant (*pFDR* < 0.05) difference between the HD network and the CG network. The asterisk (*) represents a significant (*p* < 0.025) difference between the small-world network measures from the intuitive normalization method and the traditional normalization one. The comparison of the brain networks to random networks are shown in Supplementary Figure S5. The small-worldness measures from both the intuitive and traditional normalization methods are shown in Supplementary Figure S6.

For subsequent network analyses, the correlation matrices were thresholded into both HD and CG brain networks over the entire range of sparsity, *S*, from 0 to 1. As a function of sparsity, the clustering coefficient, characteristic path length, local efficiency, and global efficiency are profiled in Fig. 5. For brain networks of the HDs and CGs, the clustering coefficients increased (Fig. 5A) but the characteristic path lengths decreased (Fig. 5B) as sparsity increased. These patterns are consistent with previous findings^36^. Moreover, clustering coefficients in the HDs (Fig. 5A) were significantly greater than in the CGs over sparsity ranges between 0.11 and 0.24 (0.0001 < *p* < 0.03), between 0.49 and 0.65 (0.004 < *p* < 0.012), and between 0.82 and 0.87 (0.001 < *p* < 0.049). Conversely, the characteristic path lengths of the brain network were significantly larger in the HDs (Fig. 5B) than in the CGs over sparsity ranges between 0.13 and 0.39 (0.001 < *p* < 0.047) and between 0.57 and 0.77 (0.007 < *p* < 0.01).

Next, we compared the HD brain network to the CG brain network. With respect to the CG brain network, as Fig. 5C shows, the normalized clustering coefficient, γ, was larger than 1, and the normalized characteristic path length, λ, was approximately 1. These features demonstrate that the HD brain network showed high levels of clustering with a low average path length compared with the CG brain network.

For the efficiency measurement, the values of local efficiency (Fig. 5D) and global efficiency (Fig. 5E) derived from the brain networks in the HDs and CGs increased as sparsity increased. The HD brain network showed significantly higher local efficiency in wide sparsity ranges (0.1 < S < 0.24, 0.022 < *p < *0.044; 0.49 < S < 0.56, 0.018 < *p* < 0.05; 0.58 < S < 0.78, 0.0001 < *p* < 0.05; 0.82 < S < 0.87, 0.001 < *p* < 0.05), and lower global efficiency (0.13 < S < 0.22, 0.0001 < *p* < 0.016; 0.25 < S < 0.39, 0.023 < *p* < 0.041) than the CG brain network. These data suggested that, compared with the CGs, the signal processing in the OFC system of the HDs may be more locally efficient but less globally efficient, particularly in the sparsity ranges from 0.13 to 0.22. It was noted that the local and the global efficiency values in the HD brain network were higher than those of the CGs in the sparsity range between approximately 0.56 and 0.78, suggesting that the concurrence may allow the HD network to balance localized processing, fault tolerance, and large-scale functional integration^37^ over this range. In the sparsity range between 0.40 and 0.46, there may be similar implications to the CG network due to the concurrence of significantly (0.018 < *p* < 0.05) higher local efficiency and considerably higher global efficiency.

Figure 5F shows that the normalized local efficiency with respect to the CG network, γ*E*, was larger than 1 and that the normalized global efficiency, λ*E,* was approximately 1. These patterns suggested that, compared with the CG brain network, the HD brain network is approximately equivalently efficient in distributed signal processing and yet more efficient in local signal processing in the OFC system. Note that there was an inconsistent pattern of global efficiency (Fig. 5E) at the sparsity threshold of 0.45, suggesting that, at values less than the threshold, the HD network having lower global efficiency had weaker “small-world” attributes^38^.

Furthermore, both the HD and CG brain networks were compared to their random networks. With respect to their random networks, the normalized values of these small-world properties (profiled in Supplementary Figure S5) suggested that both the HD and CG brain networks are small-world. The measure of network small-worldness showed a significance (*p* = 0.019) at the sparsity of 0.14 between groups. While examining the intuitive normalization method, where the HD brain network was compared to the CG brain network, and the classical normalization method, where the HD brain network was compared to random network, we found that the metrics were significantly (*pFDR* < 0.05) different at a few short sparsity ranges (0.49 < S < 0.50; 0.79 < S < 0.80; 0.82 < S < 0.83). The measures of small-world characteristics are displayed in Supplementary Figure S6.

### Regression analysis

**Figure 6:**
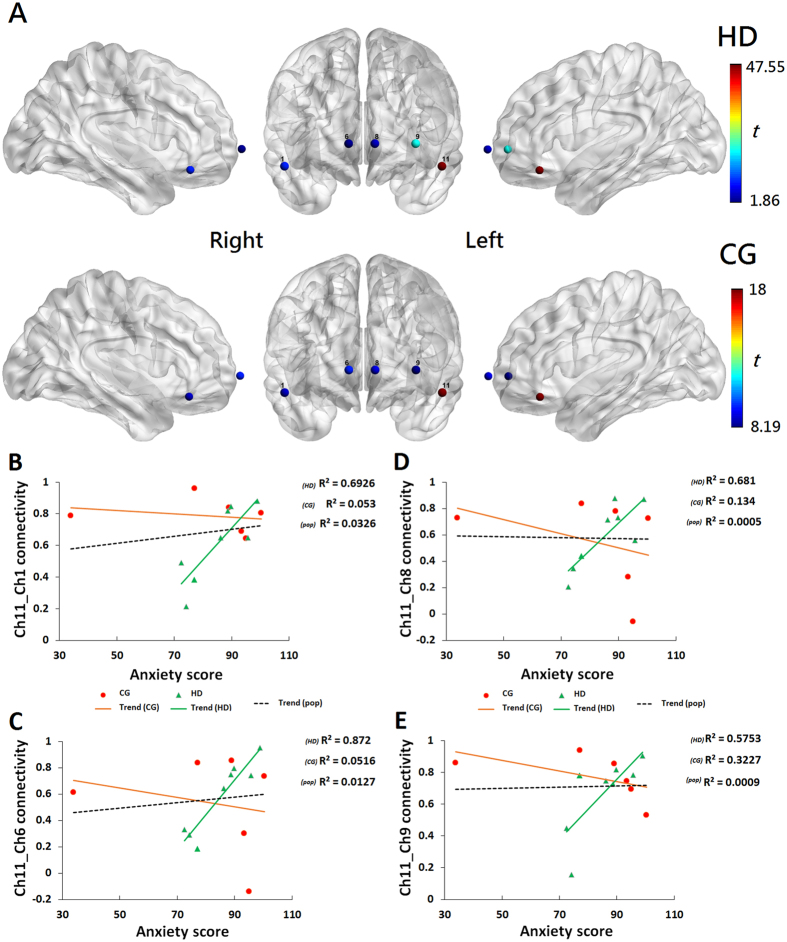
Regression analysis of the level of anxiety and functional connectivity. (**A**) (top two rows), Connectivity *T* maps between groups (N_*HD*_ = 8, N_*CG*_ = 6) are shown with respect to seed channel 11 (red). The channels are colored by their *t* values, as scaled in the color bar. The results and channels are displayed on a standardized ICBM152 brain surface from the BrainViewer. (**B**–**E**) (bottom rows), Scatter plots by group (HD: green, CG: orange) and population of the sample (pop: black) of the sample show the relationships between the STAI-anxiety score (after controlling for the covariates of age, IQ, education level, and daily cigarette consumption) and the connectivity values for channels 11 and 1 (**B)**, channels 11 and 6 (**C**), channels 11 and 8 (**D**), and channels 11 and 9 (**E**). Each regression line for the population-level sample showed significance (*pFDR* < 0.05, q-value = 0.09) and depicts the predicted model for the STAI-anxiety score and the left inferior frontal gyrus connectivity, including covariates.

Linear regression analyses were conducted to investigate the associations between the STAI scores after controlling for the covariates of age, IQ, education years, and daily consumption of cigarettes, and functional connectivity based on the seed channels (i.e., the stimuli-induced activated channels 11 and 4 from the localizer task in our study). There were significant (*pFDR* < 0.05) associations between the STAI scores and functional connectivity in the seed channel 11, but no significant association in the seed channel 4 was observed between groups. Connectivity *t*-maps are shown in Fig. 6A where the seed channel 11 is in red, and the channels with significant regression are shown and colored. Figure 6B–E show that the STAI scores after controlling for the covariates, were proportionally related to enhanced functional connectivity between the seed channel 11 (covering the left inferior frontal gyrus, orbital part), and channel 1 (covering the right middle frontal gyrus, orbital part; Fig. 6B), channel 6 (covering the right mOFC; Fig. 6C), channel 8 (covering the left mOFC; Fig. 6D), and channel 9 (covering the left superior frontal gyrus, orbital part; Fig. 6E). No significant differences between the groups in the variables age, years of education, years of smoking, daily consumption of cigarettes, and the effect of medication status were observed. Our results showed that the HDs had significantly (*p* < 0.05, Table 1) higher anxiety (STAI) scores than the CGs.

## Discussion

In this study, we utilized resting-state fNIRS measurements to characterize the cortical networks in the OFC regions in chronic heroin users in an abstinent stage. In particular, the alterations in the functional connectivity within the OFC were investigated and the question of whether anxiety is linked to the OFC networks was also examined. We discovered that the HDs who used heroin for over twenty years and who managed to stay abstinent for more than three months up to ten years exhibited manifold significant differences compared to the CGs. Overall, our findings demonstrated that the HDs showed increased interhemispheric correlations, enhanced resting-state functional connectivity, and unique network organizations in the OFC. Moreover, our group-level analysis demonstrated that the connectivity associations between the left inferior frontal gyrus seed and four OFC regions were linked to anxiety scores. We did not find evidence for associations with other seed channels in our study, nor did we find any significant differences across the participants’ demographic characteristics. Here, we consider the significant differences in the OFC networks between the HDs and the CGs from the perspective of the rsFC in turn.

With respect to the connectivity patterns of the HDs, our analyses demonstrated enhanced resting-state functional connectivity within and between the regions of interest (ROIs) in the OFC. First, our seed-based analysis estimated and detected different strength in pairwise relationships between the seeds, which were determined in our unbiased localizer^39^ task experiment, and other OFC regions. Second, our whole-brain analysis identified more connectivity patterns as indicated by increased number of edges within the region of the right lOFC (i.e., the right middle frontal gyrus and the right superior frontal gyrus, orbital part) and between the right and the left superior frontal gyri, as well as between the right and the left middle frontal gyri in the orbital part of the brain. In contrast, compared with the CG whole-brain connectivity map, the HDs showed less connectivity patterns as indicated by reduced number of edges within the mOFC. The result was consistent with our interhemispheric correlation finding where no significant intravascular events (i.e., the changes in HbO and Hb) were observed in the mOFC, which is believed to be responsible for empathic activity and is involved in emotion^40^,^41^.

Similar to the connectivity patterns, our graph-theory analysis further showed a striking phenomenon: the HDs displayed stronger connections in almost all of the channels covering the ROIs of the OFC regions than the CGs in our study. For example, the superior and the middle frontal gyri in the OFC in the left hemisphere and the superior frontal gyrus in the right hemisphere of the HDs displayed distinct overall connectivity strength.

These heterogeneous connectivity patterns within different regions of the OFC between the HDs and CGs indicated that the enhanced connectivity in these regions may manifest a neurobiological alternation in heroin addiction. Although this fNIRS-based rsFC result is novel, a number of previous “brain at rest” imaging or metabolic studies^10^,^11^,^42^,^43^,^44^,^45^ have reported the hypofunctionality of OFC in psychoactive-dependent individuals during late withdrawal (>7 days) and prolonged abstinence^46^. For example, some neuroimaging studies using fMRI and PET have reported predominantly lower activity in the mOFC and the left OFC in abstinent alcoholics^42^,^43^. Moreover, previous PET studies have shown lower metabolic rates in the right OFC and the left dorsolateral PFC in cocaine users^44^, lower dopamine receptor availability in the OFC^1^, and lower dopamine transporter density in the OFC in methamphetamine users^45^. In contrast, one perfusion MRI study observed no significant differences in the OFC in their long-abstinent methamphetamine subjects with the control group^47^. Taken together, these heterogeneous findings need further investigations to unfold the nature of the alternations in the rsFC circuitry of the OFC in drug addiction. More importantly, these studies, which used a lorazepam challenge, did not address heroin or opioid-related addiction. Interestingly, it is noted that the observed enhanced connectivity in the OFC bears an impressive resemblance to two recent advanced rsFC fMRI studies^48^,^49^, that showed stronger functional connectivity between the lOFC and the mOFC, as well as between the OFC and the nucleus accumbens (NAc) in abstinent heroin addicts than in healthy controls.

Although the functional significance of enhanced connectivity in the OFC in heroin addiction is unknown, the findings in this study may provide additional data regarding possible trajectories within the OFC regions in heroin addiction. Some theories^50^,^51^ have suggested that enhancement in the brain connectivity may be explained in the context of adapted incentive salience for the drug and drug-related stimuli. For example, if the brain area is rendered hypersensitive (i.e., sensitized), the brain system would cause pathological incentive motivation (i.e., the desire/wanting) for drugs. Correspondingly, Wilcox *et al*.^52^ reported enhanced functional connectivity between the ventromedial PFC (including a portion of the OFC) and ventral striatum (including a portion of NAc) in abstinent cocaine users. Tambini *et al*.^21^, however, suggested that the enhancement may be related to memory of recent experiences. Regardless of whether or not it is pathological, the enhanced functional connectivity implies alternations in the OFC networks in people with chronic heroin dependence. Future research is necessary to unravel the neural mechanisms of the aberrant rsFC and neural adaptions in the OFC in opioid dependence, while considering a restless connectivity over different drug-medicating cohorts.

With respect to anxiety scores, our regression analysis showed enhanced functional connectivity between the left inferior frontal gyrus seed and four OFC regions: the right middle frontal gyrus, the right and the left mOFCs, and the left superior frontal gyrus in the HDs. The left inferior frontal gyrus, which was partially covered by channel 11, is critical for inhibitory control^53^. The strong associations observed between this inhibitory region and the others may imply disruption between the OFC and amygdala linked to anxiety. One task-based neuroimaging study^40^ has supported the idea that the level of anxiety predicts the connectivity between the amygdala and medial PFC, particularly the dorsal and ventral medial PFC, which plays a critical role in executive functions. In addition to the exhibited increased functional connectivity within the OFC in the HDs who were more anxious than the CGs, our observations supported the hypotheses that there may be reduced connectivity between the amygdala and mPFC^48^, as well as the connectivity between the OFC and PFC^9^ in the individuals with heroin addiction. These assumptions help provide an explanation for the addictive phenomenon observed when an addict fails to resist impulsive desires to seek and consume drugs despite serious harmful consequences.

OFC functioning is known to be essential for social cognition processes^41^. Interestingly, the topological findings provided by our study demonstrated that the relationship between network topology and the function of social cognition is an important question that remains unsolved. When considered together with previous evidence^8^,^9^,^13^,^14^,^40^, the predictability of certain connections by monitoring the level of anxiety could be helpful in predicting relapses. It thus points to future directions; that is, investigating the neural mechanisms underlying heroin addiction in a social setting. First, is enhanced connectivity the outcome of addicts’ anxious state and traits in their environments? Second, how are the neural networks linked to social cognition, such as empathy and other processes, such as moral decisions and emotion control? Third, is enhancement the result of a therapeutic process (e.g., remaining abstinent)? The answers to these questions could be tremendously helpful in the development of psycho-social-behavioral intervention and treatment programs.

As current advances in rs-fNIRS have been shown to be a valid and supportive tool for investigating the rsFC in normal and pathological conditions^16^,^17^,^54^, our study also validated fNIRS as an additional neuroimaging method available to examine cortical mechanisms underlying addiction. Unlike fMRIs, fNIRS has higher temporal resolution, and thus it allows precise quantification of the network parameters and measures when and which OFC regions were consuming oxygen^23^,^24^. Using fNIRS, perhaps another remarkable finding was that a small-worldness architecture was quantified and observed in the brain networks of the HDs and CGs, providing a profile of clustering coefficients and characteristic path lengths over an entire range of sparsity thresholds. Moreover, our exploratory work produced evidence that the HD network was “small-world” with respect to the CGs, suggesting that the HD brain network acquires non-control and non-random construction. Though, the new normalization method is experimental, and thus the results from the method should be read with caution and should be served as additional information to the ones from the traditional method.

The major findings examined above were primarily based on the abnormal resting state observed in abstinent heroin addicts and the controls using fNIRS. Additional characteristics of functional connectivity in the circuitry associated with addiction, such as reward, anticipation, emotion, and environmental stressors, such as anxiety, remain to be elucidated. Due to the small sizes of our sample without strict control of the alpha-error inflation resulting from manifold analyses and the control for multiple covariates, questions remain regarding to what extent the present findings are linked to the abnormality of brain functions in drug abusers and to what degree the current results are stable and reliable. Therefore, findings presented in this study must be treated with caution until results are confirmed in a larger sample. Forthcoming fNIRS-based work on the rsFC in the remainder of the brain regions involved in opioid dependence, including dependence on prescribed opiates, such as morphine, oxycodone, and fentanyl, is needed to provide a more definitive and meticulous explanation of the current study. Moreover, future studies involving different treatment-/intervention-programs for opioid dependence and behavioral tasks are needed to finalize conclusions, such as examining the task-based functional connectivity and the link between the neuroimaging results and behavioral data, and defining the influences of other drugs available in treatment, such as methadone, buprenorphine, naloxone, and naltrexone, on functional connectivity. We observed that the year of smoking was correlated with anxiety scores, and thus co-dependence on other substances, such as nicotine, and comorbid conditions, such as anxiety, should be carefully taken into account in future studies.

In summary, we have presented important exploratory work using fNIRS to study alternations of OFC functional connectivity in heroin addiction. We have demonstrated evidence of enhanced functional connectivity across OFC regions for abstinent heroin abusers. Although preliminary, the findings contribute new data to an emerging literature on the neuropsychological basis of addiction in the OFC and the resting-state mechanism underlying opioid dependence.

## Methods

### Participants

From May 5, 2015 to September 15, 2015, eight abstinent HD individuals were recruited from a local drug rehabilitation treatment center in Macao SAR and seven control subjects from the local community were enrolled with demographic factors matching those of the HDs (i.e., age, education level, IQ, and years of smoking; Table 1). All the subjects were smokers. In order to control for the effects of nicotine, daily cigarette consumption of the CGs was matched with the HDs. The protocol was approved by the Medial Ethics Committee of the University of Macau. The experiments were performed in accordance with the relevant guidelines and regulations in the latest version of the Declaration of Helsinki. After being providing with an informed description of the study procedures, all the participants signed informed consent forms prior to the experiments.

The inclusion criteria for all the participants were as follows: (1) aged 18–65 years old, (2) right-handed, (3) Cantonese speakers, and (4) normal or corrected-to-normal vision. The exclusion criteria for all the participants were as follows: (1) history of neurological illness or psychiatric disorder other than heroin and nicotine dependence, (2) reported additional use of psychoactive substances at least 72 hours before any measurement, and (3) history of head trauma or brain injury.

All the HD subjects had a history of more-than-20-years of heroin abuse. Local medical doctors confirmed the diagnoses of drug addiction. All the participants were required to complete a preclinical interview performed by a local psychological counselor prior to inclusion in the study. The Structured Clinical Interview for DSM-IV (SCID) was used in the interview to verify opioid dependence, and the State-Trait Anxiety Inventory (STAI) was adopted to measure the degree of anxiety. None of the HD subjects had received any formal psychotherapeutic training. Information on the subjects’ demographic characteristics is provided in Table 1. After the preclinical interview, a urine sample was collected from each subject for mandatory drug screening to ensure that the subject had not taken any psychoactive drugs other than heroin and methadone before any assessments or recordings. Subjects were compensated with a small fee upon their completion of the experiment. The medication and substance-use status of the subjects is provided in Supplementary Information S7.

### Experimental design

The subjects were instructed to sit comfortably on a chair in a quiet and dim room. All the subjects underwent two sessions of fNIRS recordings of neural activities of the OFC. The first session was an 11-min resting-state recording followed by the second session, an approximately 3-minute gambling localizer task.

During the resting-state session, the subjects were asked to close their eyes, relax their muscles and mind, think of nothing, remain still and make the least possible amount of motion, but to stay awake and alert. After the resting-state recording, the subjects were instructed to open their eyes to conduct a gambling task to retrain the OFC regions for further rsFC analysis.

In the rsFC analysis, four functional connectivity methods were utilized to generate regional brain networks related to drug addiction, which included: (1) interhemispheric correlation analysis, (2) functional connectivity measurements generated by seed-based and whole-brain correlation analyses, (3) graph-theory network analysis, and (4) linear regression analysis (for experimental design details, see Supplementary Figure S1).

### fNIRS data acquisition

**Figure 7:**
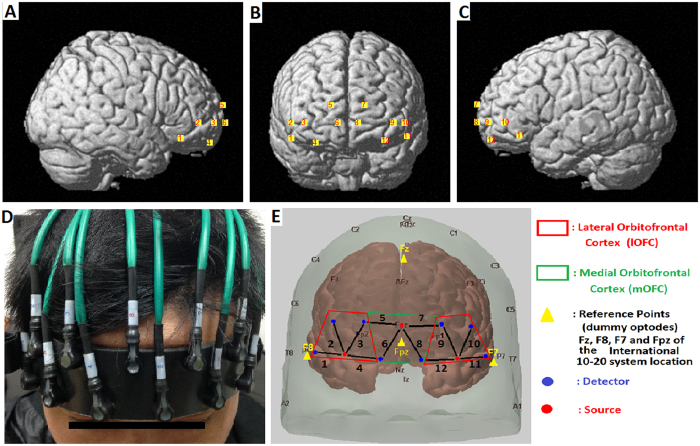
The position of the 12 channels on both hemispheres covering the orbitofrontal cortex. (**A**–**C**) Channels were numbered on the right side, frontal and left side of the brain, respectively. (**D**) Front view of the probe arrangement. (**E**) The location of the optodes (3 sources: red dots, 8 detectors: blue dots). Each source and each detector were 30 mm apart. A black line connecting a detector and a source represents a measurement channel with a number. There were 6 channels from each side of the brain: 2 channels covering the medial OFC (right hemisphere: channels 5 and 6; left hemisphere: channels 7 and 8) and, 4 channels covering the lateral OFC (right hemisphere: channels 1–4; left hemisphere: channels 9–12). The medial OFC (mOFC) was bounded with the green line and lateral OFC (lOFC) with the red line. The probe was projected on the scalp with the anchor points in the yellow triangle of Fz, F7, and F8 based on the International 10–20 system. The source in the middle of the probe array was located on the Fpz. The locations of the optodes in the both hemispheres were anatomically symmetrical.

The experiments were performed using a continuous wave (CW) fNIRS instrument with three sources and eight detectors (CW6 fNIRS system; TechEn Inc, Milford, MA), as shown in Fig. 7. In this system, two CW lights at wavelengths of 690 nm and 830 nm were emitted at each source fiber, which provided sensitive detection of changes in both the HbO and Hb concentrations in the human brain. The configuration of the source-detector pairs is shown in Fig. 7, in which the sources and detectors were connected by optical fibers on the scalp to generate twelve fNIRS measurement channels covering the bilateral and medial OFC regions, which were positioned accurately based on the 10–20 system^55^. Each set of one source and four detectors covered one particular OFC, resulting in four ROIs; i.e., the right lOFC, the left lOFC, the right mOFC, and the left mOFC (see Supplementary Table S2). The MNI coordinates of the channels were estimated using Homer2 (www.homer-fnirs.org/documentation) and its AtlasViewerGUI^56^ with the widely adopted automated anatomical labeling (AAL). The three-dimensional locations of the fNIRS probes were also visualized with the NIRS-SPM software (http://bispl.weebly.com/nirs-spm.html#/). The source-detector distance was 30 mm, measuring the brain activity of the cerebral cortex with a penetration depth of 2–3 cm. The sampling rate of our fNIRS system was 50 Hz. Using MATLAB (MathWorks, Inc., Natick, MA, USA) and BrainNet Viewer (https://www.nitrc.org/projects/bnv), statistical outcomes for each channel were shown on the surface of a normalized and a standardized brain^57^.

### Preprocessing data

All the resting-state fNIRS data were preprocessed using Homer2. For the resting-state data, the first 2-minute measurements were eliminated to generate relatively steady signals and rule out potential effects of unstable signals before rsFC analysis^36^,^54^,^58^. For the localized task data, the first 30-second recordings were eliminated for the recording signals to reach a steady rate.

The following preprocessing procedures^36^,^54^ were performed for all the fNIRS data: (1) Conversion of optical density measurements to concentration changes in HbO and Hb at different time points based on the modified Beer-Lambert Law^59^; (2) Bandpass filtering (resting-state^27^: 0.01 < f_r_ < 0.1; task^60^: 0.012 < f_t_ < 0.18 in Hz); (3) Detrending; and (4) Motion correction using the spline interpolation method^61^ and a correlation-based signal improvement (CBSI) method^62^.

To ensure high-quality fNIRS data for the rsFC network analyses, motion-induced artifacts and low signal-to-noise ratios (SNRs) were examined with an open-source package, FC_NIRS (https://www.nitrc.org/projects/fcnirs/). Any data with low average signal intensity over five standard deviations of the HbO and Hb concentrations over time (i.e., lower SNRs) were considered to result from poor contact between the optode and scalp and thus were excluded from the rsFC and network analyses. One CG subject’s channel 11 was excluded.

For task-based fNIRS data, the HbO and Hb signals from each channel across all the subjects were extracted for statistical analysis to determine the seed channels for the rsFC analysis.

For rsFC and graph analyses, two thresholding strategies were applied: (1) a single threshold representing the absolute connectivity strength was chosen, and (2) the entire range of relative sparsity thresholds and average graph property values over the range were studied. By applying these thresholding strategies together^63^, we hoped to provide a comprehensive way to examine the network organizations of both groups.

### Interhemispheric correlation analysis

We used interhemispheric correlations to demonstrate the long-range connectivity of the right and left lateral OFC (lOFC) and medial OFC (mOFC) in both the HDs and CGs. All ROIs within the OFC were investigated for local connectivity between the right and left hemispheres (see Supplementary Table S2). The typical patterns observed in the cortical blood flow during brain activation^34^ are an increase in HbO and a decrease in Hb. As the regions covered by the channels were very close to each other anatomically, we assume that the long-range activation plays a role in the connectivity because the interhemispheric correlations analysis is designed to study a long-distance brain connectivity that is likely connected to the intravascular (HbO and Hb) events^34^ between hemispheres. Therefore both HbO and Hb were considered for the interhemispheric analysis. Pearson’s correlation coefficient between the time course of every channel and the matching symmetrical measurement channel in the other hemisphere from the selected ROI in the OFC system (overall, lOFC and mOFC) was calculated, and the mean correlation coefficient was then calculated for all related channels in each ROI.

### Functional connectivity measurements: seed-based and whole-brain correlation analyses

To analyze the rsFC of the HDs and CGs, we used two approaches: (1) a seed-based correlation analysis and (2) a whole-brain correlation analysis. The former approach was used in order to estimate and detect the strength of pairwise relationships between a predefined seed channel and other channels covering other OFC regions. This allowed us to identify intrinsic functional connectivity in different OFC region systems. The second approach aimed to compute a functional connectivity map between any two channel pairs in the entire OFC system, where the measurement channels represented nodes, and the functional connections with correlation coefficients greater than a predefined threshold were considered edges^64^. The change in HbO concentration is recognized to be the most responsive indicator of differences in cortical blood flow^34^. We believe that HbO signals would be responsive enough to indicate changes in the local activity in the regions covered by the channels that were not far apart from each other as abovementioned. For simplicity, HbO was therefore considered for the remainder of the analyses. The Hb data was served as a reference only when needed.

The gambling localizer task data were processed using a general linear model to estimate the task-induced response of each channel from each subject. The run averages of the normalized HbO data were first calculated for each channel, and then grand average results across all subjects by group were calculated and analyzed. A statistical *t*-test was performed to infer which channel was most significantly (*p* < 0.05) activated by the localizer task (see Supplementary Figure S3). The channel(s) and the corresponding channel(s) on the other hemisphere were then defined as the seeds for the rsFC analysis.

For the resting-state data, functional connectivity was then constructed based on the seed-based and whole-brain correlation methods; thus, functional connectivity matrices and maps were first generated from group adjacencies computed from the individual adjacencies for the brain network analysis. For both the seed-based and whole-brain correlation methods, Pearson’s correlation was used to weigh the strength of functional connectivity of the pairwise relationships between the selected seed channel and other channels and between any two node pairs within the entire OFC system in our study. Prior to any statistical analysis, the correlation values were converted to Fisher z-values using the Fisher z-transformation^65^. Thus, the distribution of the Z to R correlation coefficients pairwise in the OFC can be observed in two groups using the seed-base analysis, and the neural network generated *within* the entire OFC regions between the groups can be explored using the whole-brain approach.

### Graph-theory network analysis

To quantify rsFC for complex network analysis, powerful graph-theory approach^27^ was adopted to compare the fNIRS-based topological properties of the brain networks between the HDs and the CGs. Using the free software, GRETNA^66^ (http://www.nitrc.org/projects/gretna), the preprocessed fNIRS data with N nodes were used to form an N-by-N correlation matrix in which each value represented the connectivity strength. The network measures were categorized into global and nodal metrics. The global metrics used included small-world parameters^67^, local efficiency and global efficiency^37^. In the global network analysis, two important small-world parameters, (1) clustering coefficient and (2) characteristic path length, were then calculated and compared with the normalized small-world parameters between the brain networks of both HDs and CGs. These global network metrics provided a global topological framework of the whole-brain network, taking into account all-channel networks in the OFC system. In the nodal network analysis, the nodal degree strength of each channel and efficiency^68^ was examined. These nodal metrics provided information on the regional patterns of the functional brain network.

Traditionally, a small-world graph is one where the clustering coefficient of a brain network is higher than that of a random network and where the path-length is characteristically similar to that of a random graph^38^. Thus, normalization of clustering coefficients and path-length are traditionally computed with respect to random networks. However, it is not uncommon to compare the brain networks of subject groups to detect connectivity abnormalities in psychiatric disorders^63^,^69^,^70^. Intuitively, in addition to this tradition, we wanted to explore whether the HD network is “small-world” with respect to the CG network; thus, small-worldness was computed by comparing the HDs with the CGs (i.e., γ = clustering coefficient in the HDs ÷ clustering coefficient in the CGs; λ = characteristic path length in the HDs ÷ characteristic path length in the CGs; γ*E* = local efficiency in the HDs ÷ local efficiency in the CGs; λ*E* = global efficiency in the HDs ÷ global efficiency in the CGs). As such, this preliminary investigation may provide a direct metric to access “small-worldness” in observed real-world networks^63^, which are neither uniformly random nor ordered^64^. A two-sample *t*-test (α = 0.05) followed by a correction approach with a false discovery rate (FDR) was applied^66^ to examine whether the HD brain network acquires significantly non-control and non-random construction. Further, the small-world network measure^38^,^71^ will be used to characterize differences between the metrics of the traditionally normalized coefficients (i.e., HD_*net*_/Random_*net*_ and CG_*net*_/Random_*net*_) and compare with the metric of the intuitively normalized ones (i.e., HD_*net*_/CG_*net*_).

### Regression analyses for anxiety scores and seed-based functional connectivity

Linear regression analysis was conducted to examine the link between anxiety severity (STAI scores) and functional connectivity for all seed channels. Multiple regression analysis was performed for all significant results to statistically account for potential differences in age, years of education, IQ, STAI scores, daily cigarette consumption, years of smoking, and the effect of methadone treatment. The seed-based functional connectivity presented in the correlation coefficient R to Z values between all the selected seed channels and the remainder of the channels were examined with the anxiety scores after controlling for the covariates. To address the issue of multiple comparisons, all results were controlled using the Benjamini–Hochberg procedure^72^ at an FDR < 10%.

## Additional Information

**How to cite this article**: Ieong, H.F. and Yuan, Z. Abnormal resting-state functional connectivity in the orbitofrontal cortex of heroin users and its relationship with anxiety: a pilot fNIRS study. *Sci. Rep.* **7**, 46522; doi: 10.1038/srep46522 (2017).

**Publisher's note:** Springer Nature remains neutral with regard to jurisdictional claims in published maps and institutional affiliations.

## Supplementary Material

Supplementary Materials

## Figures and Tables

**Table 1:** Demographic characteristics.

Variable	All participants (n = 15)		Heroin-dependent Group (HD) (n = 8)		Control Group (CG) (n = 7)		*p*
	mean	s.d.	mean	s.d.	mean	s.d.	
Age (years)	45.7	6.8	47.6	6.1	45.7	6.8	0.58
Education (years)	6.4	2.7	5.9	2.8	7.0	2.4	0.43
IQ	83.0	10.5	82.9	11.4	83.1	9.3	0.97
Cigarettes (mg/per day)	21.5	10.3	22.8	9.7	20.0	10.7	0.61
History of smoking (years)	31.3	7.4	33.6	6.7	28.7	7.3	0.89
Anxiety	85.5	21.9	95.4	22.1	74.1	15.2	0.047^*^
Duration of heroin use (years)	N/A	N/A	29.0	9.7	N/A	N/A	N/A
Duration of heroin abstinence (years)	N/A	N/A	2.39	3.4	N/A	N/A	N/A
Average heroin dosage (g/day)	N/A	N/A	0.6	0.4	N/A	N/A	N/A
Methadone treatment dosage (mg)	N/A	N/A	30.0	26.9	N/A	N/A	N/A

The *p* values are reported for a two-sample *t* test (for age, years of education, IQ, daily cigarette consumption, years of smoking, and level of anxiety) comparing abstinent heroin-dependent subjects (HDs) with the controls (CGs). IQ was assessed with the Wechsler Adult Intelligence Scale version 4. Anxiety scores were scaled with the adult State-Trait Anxiety Inventory (STAI^73^). Abbreviation: Standard deviation (s.d.). *p < 0.05.
