# Supplementary material for: Abnormal resting-state functional connectivity in the orbitofrontal cortex of heroin users and its relationship with anxiety: a pilot fNIRS study
Source: Sci Rep. 2017 Apr 19;7:46522. doi: 10.1038/srep46522 (PMC5395928; doi:10.1038/srep46522)
Supplement: Supplementary Materials [file srep46522-s1.pdf]

Abnormal resting-state functional connectivity in the orbitofrontal cortex of heroin users and its relationship with anxiety: a pilot fNIRS study

Hada Fong-ha Ieong[1], Zhen Yuan[1,*]

[1] University of Macau, Faculty of Health Sciences, Bioimaging Core, Macau SAR, 99999, China

* zhenyuan@umac.mo

Contents

Supplementary Figure S1 2

Supplementary Table S2 3

Supplementary Figure S3 4

Supplementary Table S4 5

Supplementary Figure S5 6

Supplementary Figure S6 7

Supplementary Information S7 8

References 9

Supplementary Figure S1.

Experimental representation for the fNIRS-based rsFC analysis. ***A***, The resting-state fNIRS recording session is followed by a gambling task recording session. During the task session (G, gambling), a subject was told that a number was hidden behind a card located in the center of a screen. The card would be displayed for three seconds, and the subject's task was to predict whether the number behind the card was bigger or smaller than five. At the end, the subject was told the number of corrections. The purpose of the gambling task was to simulate real-life anticipating and decision making of a subject who was asked to make the prediction. The HbO and Hb signals from the three-second fNIRS recordings data were extracted and analyzed to determine the stimuli-induced activation seed channel(s) for the rsFC analysis. The gambling task, adopted from The Human Connectome Project[1], was presented by E-Prime software (E-Prime 2.0, Psychology Software Tools, Inc., Pittsburgh, PA, USA). ***B*,** The fNIRS data was analyzed accordingly from the corresponding recording session from the top in ***A***.

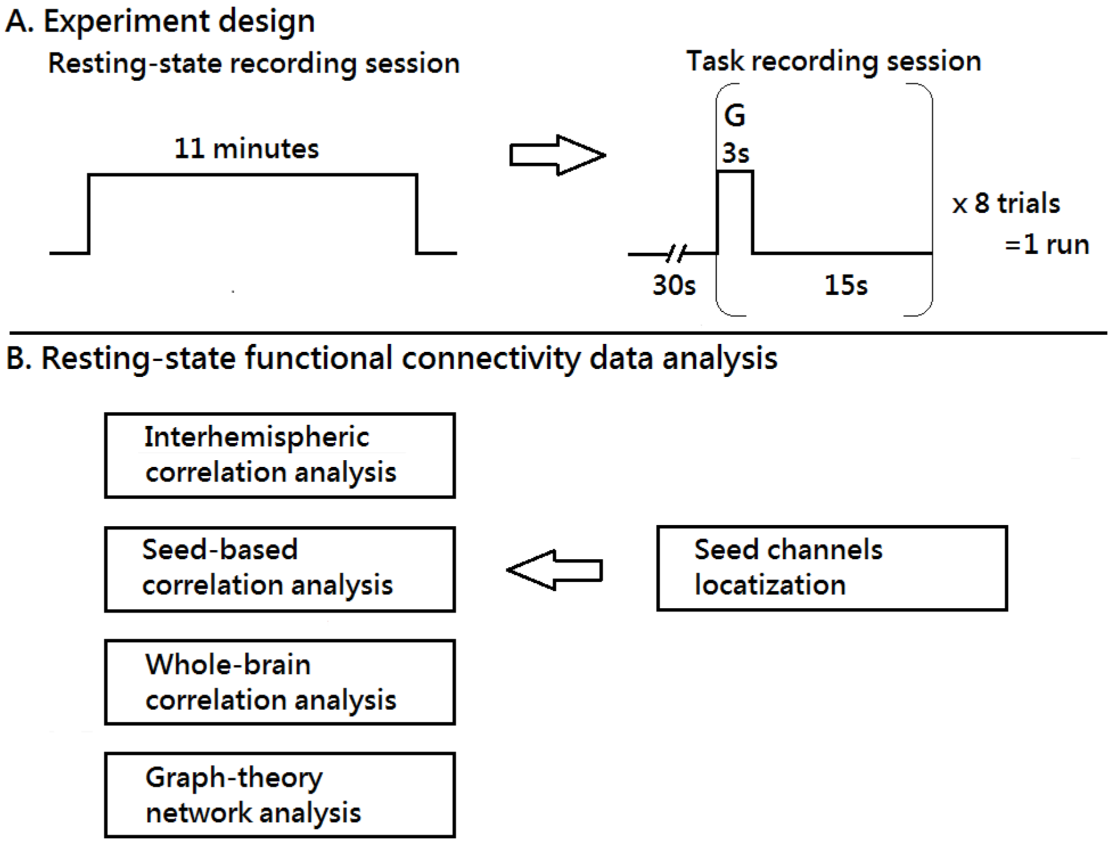

Supplementary Table S2.

Summary of the four-ROIs and their associated seed channels and the MNI coordinates. Channel coordinates were generated using the AtlasViewerGUI[2] program in Homer 2. Automated anatomical labeling (AAL) is applied.

ROIs	Channel	Coordinates (MNI)			Label (AAL)
**Right lOFC**	1	92	153	196	Right middle frontal gyrus, orbital part
	2	102	150	197	Right middle frontal gyrus, orbital part
	3	108	155	205	Right superior frontal gyrus, orbital part
	4	113	161	207	Right superior frontal gyrus, orbital part
**Right mOFC**	5	120	150	213	Right medial orbitofrontal cortex
	6	124	160	214	Right medial orbitofrontal cortex
**Left mOFC**	7	144	149	214	Left medial orbitofrontal cortex
	8	138	156	211	Left medial orbitofrontal cortex
**Left lOFC**	9	154	156	209	Left superior frontal gyrus, orbital part
	10	167	156	205	Left middle frontal gyrus, orbital part
	11	164	156	198	Left inferior frontal gyrus, pars orbitalis
	12	147	158	204	Left superior frontal gyrus, orbital part

Supplementary Figure S3.

Localizer task-induced activation map. ***A*****,** Statistical population-level *t*-map indicating the channels (i.e., channels 11 and 4) robustly activated in the gambling localizer task ($p < 0.05$). All the channels are numbered. Color bar indicates the *t*-values. ***B*-*C***, Resting-state HbO signals obtained from the OFC seed channels 11 and 4 in the HDs and the CGs, respectively. Two-second resting-state time courses are displayed.

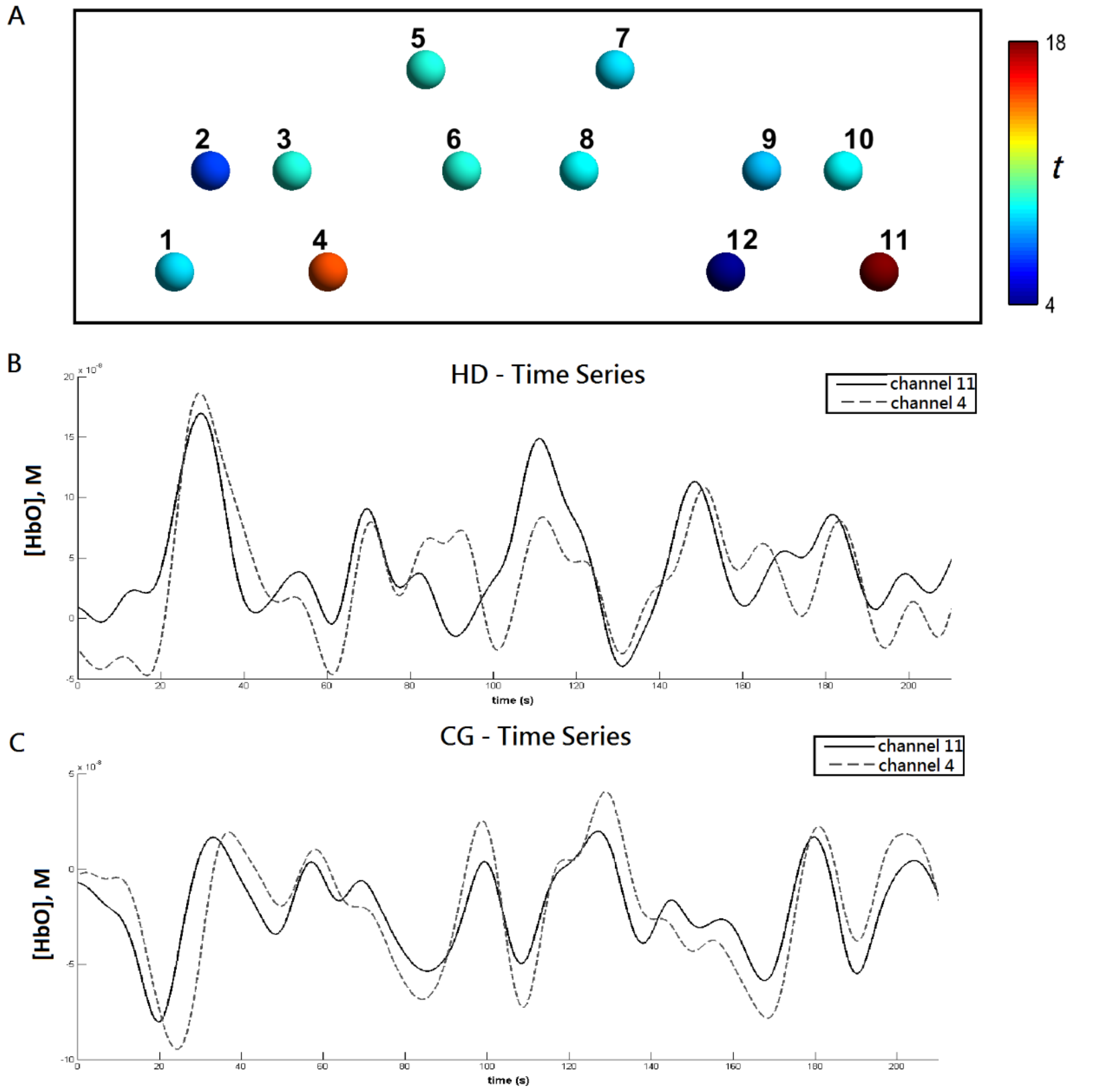

Supplementary Table S4.

Table of connectivity strength by channel. Connectivity strength, σ, along with the standard error of the mean (s.e.m.), indicates the average connectivity of a channel in the HD and CG networks in the OFC system.

**Channel/node**	**HDs (n = 8)**		**CGs (n = 7)**		*p*
	σ	**s.e.m.**	σ	**s.e.m.**	
1	0.83	0.031	0.71	0.035	0.014
2	0.81	0.024	0.74	0.028	0.042
3	0.88	0.012	0.75	0.024	0.0001
4	0.83	0.028	0.73	0.030	0.029
5	0.78	0.032	0.67	0.041	0.044
6	0.85	0.019	0.75	0.028	0.009
7	0.76	0.037	0.68	0.038	0.115
8	0.84	0.020	0.77	0.022	0.034
9	0.88	0.009	0.74	0.021	< 0.0001
10	0.84	0.017	0.67	0.024	< 0.0001
11	0.80	0.026	0.69 [a]	0.028 [a]	0.008 [a]
12	0.83	0.029	0.74	0.032	0.039

- [a] N_{CG} = 6, the channel was excluded due to a poor contact between the optodes.

Supplementary Figure S5.

Comparison of the HD and CG brain networks to their random networks. The normalized values of small-world properties and network efficiency of HbO-based networks of the HDs and CGs with respect to their random networks are profiled as a function of sparsity. The 100 matched random networks were generated by the Markov-chain algorithm[3,4] as a function of sparsity, preserving the same number of nodes and edges and degree distribution with the respect to the real brain network of both groups. ***A*-*B*** (first column), The normalized values of clustering coefficient (γ_C), path length (λ_C), local efficiency, (γE_C), and global efficiency (λE_C) of the CG network are plotted in green over the range of sparsity thresholds from 0.05 to 1, respectively. The normalized values of the λ_C and λE_C in the CGs share similar patterns, resulting in almost all the values of γ_C and $\gamma E_C > 1$, and λ_C and λE_C = ~1. These findings may indicate that the CG network has small-world characteristics in the OFC systems. Similarity, ***C*-*D*** (second column)**,** present the normalized values of γ_H , λ_H , γE_H , and λE_H of the HD network respectively. Almost all the values of γ_H and $\gamma E_H > 1$, and λ_H and λE_H are = ~1, suggesting that the HD network is also small-world. The asterisk (*) represents a significant ($p < 0.05/2 = 0.025$; 2 statistical tests) difference between the small-world measures of the HDs and CGs at Sparsity = 0.14.

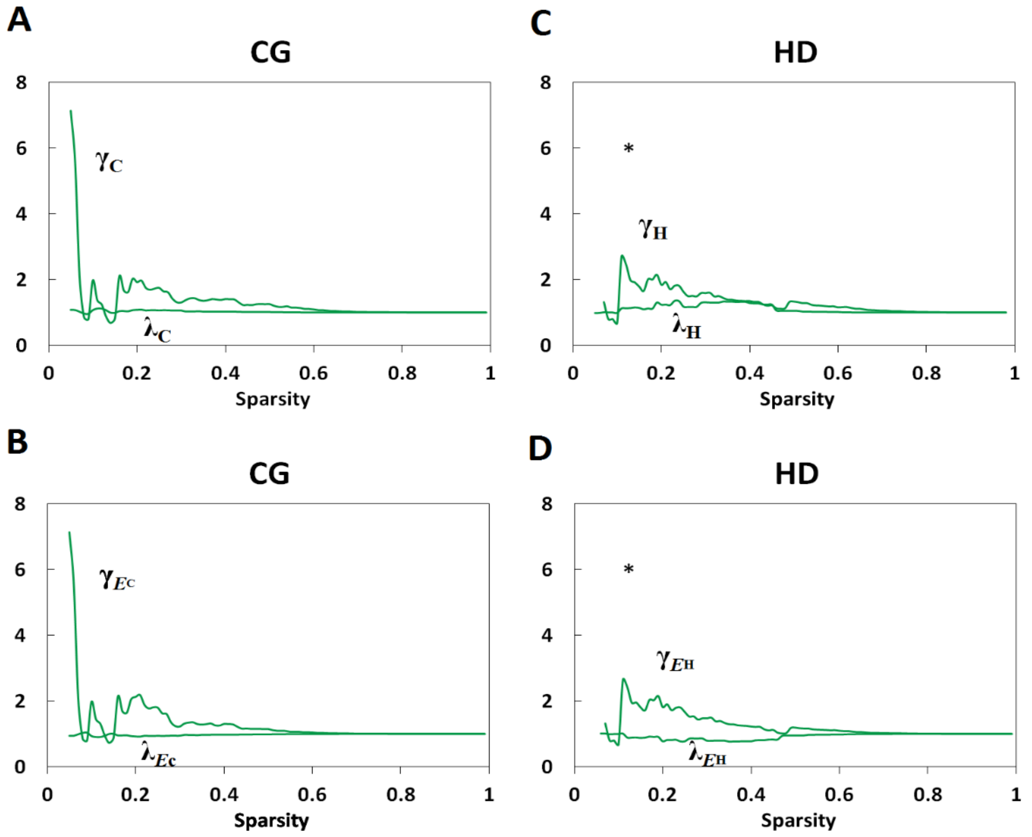

Supplementary Figure S6.

Scatter plot of small-worldness network measures. The mean *Sigma* values are plotted as a function of sparsity in the range between 0 and 1. The blue displays the small-world characteristics of the HD brain network with respect to the random network. The red displays the characteristics of the CG brain network with respect to the random network. The green displays the HD brain network with respect to the CG brain network. The error bars show the standard deviations.

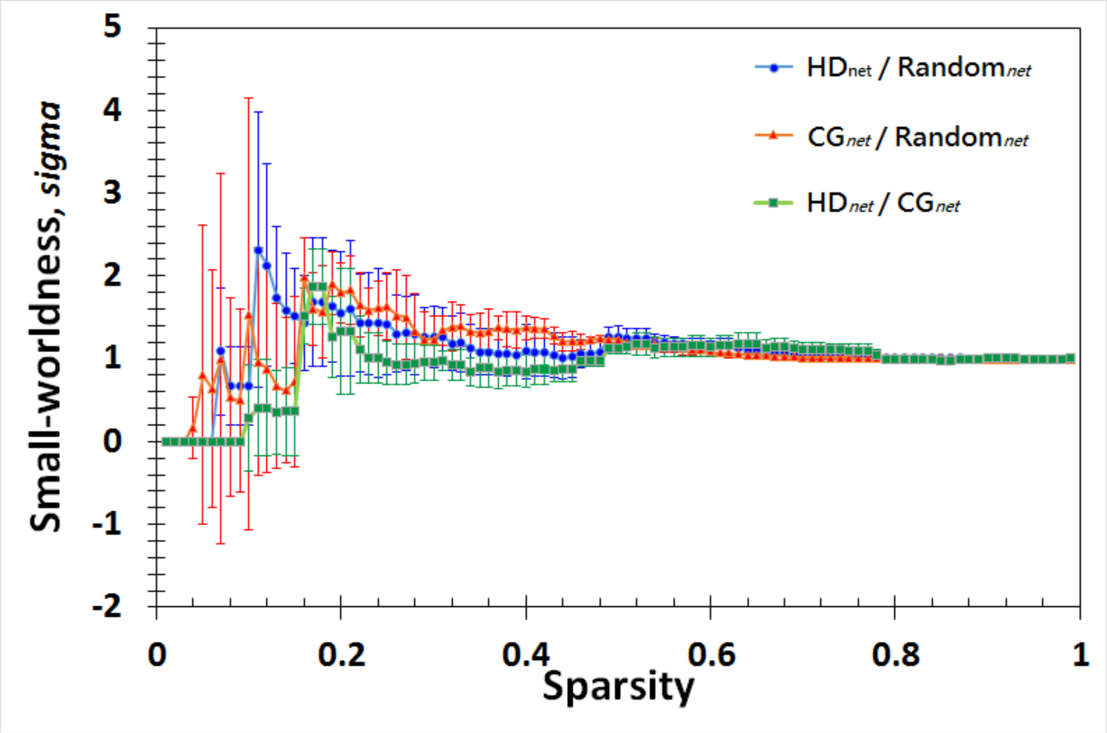

Supplementary Information S7.

Medication and substance-use status of subjects at the time of the study. Two HD subjects were undergoing the methadone (40 mg per day) maintenance treatment. One HD subject was taking simvastatin (20 mg). One CG subject reported to take trazodona (50 mg) in life time but reported not taking it during the period of the study. The urine screening reports of all the participants showed negative results (the absence) of amphetamine, oxycodone, heroin, morphine, codeine, tetrahydrocannabinol, cocaine, benzodiazepine, diazepam, and their metabolites. All the participants were chronic and heavy smokers. Our result showed that the year of smoking was significantly correlated with the STAI scores on a population level of the sample (R^2 = 0.550, p = 0.021, N = 15).

References

1 Barch, D. M. *et al.* Function in the human connectome: task-fMRI and individual differences in behavior. *Neuroimage* **80**, 169-189, doi:10.1016/j.neuroimage.2013.05.033 (2013).

2 Aasted, C. M. *et al.* Anatomical guidance for functional near-infrared spectroscopy: AtlasViewer tutorial. *Neurophotonics* **2**, 020801-020801 (2015).

3 Maslov, S. & Sneppen, K. Specificity and stability in topology of protein networks. *Science* **296**, 910-913 (2002).

4 Sporns, O. & Zwi, J. D. The small world of the cerebral cortex. *Neuroinformatics* **2**, 145-162 (2004).

5 Braun, U., Muldoon, S. F. & Bassett, D. S. On human brain networks in health and disease. *eLS* (2009).
